# Characterizing cognitive aging of recognition memory and related processes in animal models and in humans

**DOI:** 10.3389/fnagi.2012.00015

**Published:** 2012-09-12

**Authors:** Sara N. Burke, Lee Ryan, Carol A. Barnes

**Affiliations:** ^1^Evelyn F. McKnight Brain Institute, University of ArizonaTucson, AZ, USA; ^2^Memory and Aging, ARL Division of Neural Systems, University of ArizonaTucson, AZ, USA; ^3^Departments of Psychology and Neurology, University of ArizonaTucson, AZ, USA

**Keywords:** medial temporal lobe, monkey, perception, perirhinal cortex, rat

## Abstract

Analyses of complex behaviors across the lifespan of animals can reveal the brain regions that are impacted by the normal aging process, thereby, elucidating potential therapeutic targets. Recent data from rats, monkeys, and humans converge, all indicating that recognition memory and complex visual perception are impaired in advanced age. These cognitive processes are also disrupted in animals with lesions of the perirhinal cortex, indicating that the the functional integrity of this structure is disrupted in old age. This current review summarizes these data, and highlights current methodologies for assessing perirhinal cortex-dependent behaviors across the lifespan.

## Object recognition and the perirhinal cortex: animal experiments

The ability to discriminate novel stimuli from ones that have been previously encountered is a basic cognitive skill necessary for an animal's survival. Moreover, this facility is an integral aspect of memory encoding that declines during normal aging (e.g., Moss et al., 1988; Bachevalier et al., 1991; Head et al., 1995; Herndon et al., 1997; Pihlajamaki et al., 2004; de Lima et al., 2005; Pitsikas et al., 2005; Pieta Dias et al., 2007; Insel et al., 2008; Burke et al., 2010). For these reasons, tasks that examine an animal's ability to recognize a familiar stimulus have a long history (Harlow, 1944), and researchers have spent over five decades trying determine the brain structures that contribute to recognition. Interestingly, the results of many experiments are ambiguous regarding whether or not judgments of recognition require an intact hippocampus (e.g., Baxter and Murray, 2001a; Clark et al., 2001; Zola and Squire, 2001; Broadbent et al., 2004). In contrast to the hippocampus, lesion experiments in rats (Mumby and Pinel, 1994; Ennaceur et al., 1996; Ennaceur and Aggleton, 1997; Bussey et al., 2000; Kesner et al., 2001; Gaffan et al., 2004; Prusky et al., 2004; Winters and Bussey, 2005) and monkeys (Meunier et al., 1993; Buffalo et al., 1999, 2000; Malkova et al., 2001) have unequivocally demonstrated that the perirhinal cortex is necessary for object recognition.

Multiple tasks have been designed to measure object recognition. The delayed-non-matching-to-sample (DNMS) task, which was first designed for monkeys (Mishkin and Delacour, 1975), can also be adapted to test a rat's ability to discriminate between a sample stimulus and a novel stimulus. In this task, the animal must displace a single sample object to receive a food reward. After a variable delay, the animal is presented with two objects, the original sample object and a novel object. If the animal displaces the novel object, it receives a food reward and this is recorded as a correct trial. The trial is incorrect if the animal displaces the sample object and it does not receive a reward (Mishkin and Delacour, 1975; Mumby et al., 1990, 1992; Mumby and Pinel, 1994). Lesions of the perirhinal cortex in monkeys produce impairments on the DNMS task when trial-unique stimuli are used (Meunier et al., 1993; Buffalo et al., 1999, 2000; Malkova et al., 2001). When a small set of familiar stimuli are used, however, perirhinal cortex lesions in monkeys do not produce a significant impairment on the DNMS task (Eacott et al., 1994). Additionally, rats with perirhinal cortex lesions perform worse on the DNMS task relative to control animals (Mumby and Pinel, 1994).

Importantly, normal aging is not comparable to a perirhinal cortical lesion as there is no significant loss of neurons in this brain structure over the lifespan (Rapp et al., 2002). Neurochemical studies, however, have indicated that several different amino acids, including glutamate (Liu et al., 2009; Rushaidhi et al., 2012), and polyamines necessary for normal cellular function (Liu et al., 2008) show altered levels in the perirhinal of aged rats compared to young animals. Moreover, there is evidence that aged rats have reduced levels of the calcium-binding protein calbindin in the perirhinal cortex, which suggests potential impairments in calcium homeostasis within this brain region (Moyer et al., 2011). Together these data suggest that the perirhinal cortex is among the structures adversely affected by the process of normal aging. Several studies have measured the effect of advanced age on DNMS task performance and found that aged monkeys are also impaired (e.g., Moss et al., 1988; Rapp and Amaral, 1991; Shamy et al., 2006), which could indicate functional alterations of perirhinal cortex circuits that support the recognition of visual stimuli. The DNMS task has never been used to measure object recognition in aged rats, but the spatial variant of this task [delayed non-match-to-position (DNMP)] has been used to demonstrate that old rats do not effectively discriminate between the sample and the novel position when compared to young animals (Dunnett et al., 1988). Although perirhinal lesions produce similar deficits on the DNMP task in rats (Wiig and Burwell, 1998), this age-associated impairment is difficult to interpret for several reasons. First, only two different stimuli are generally used in the DNMP task (left lever vs. right lever); therefore, by virtue of this design, the DNMP task cannot use trial unique stimuli, which may limit the requirement of an intact perirhinal cortex for normal performance. Additionally, this task is spatial, and the perirhinal cortex is only required for spatial tasks when the testing environment is rich in visual cues (for review, see Aggleton et al., 2004). For these reasons the DNMP task is not optimal for assessing recognition deficits that might arise from possible age-associated alterations in the perirhinal cortex.

Although it is evident that the perirhinal cortex is critical for stimulus recognition, the primary role of the perirhinal cortex in cognition is still debated. Specifically, some researchers have argued that the perirhinal cortex participates in a medial temporal lobe memory system but does not significantly contribute to perception (e.g., Squire and Zola-Morgan, 1991; Buffalo et al., 1998; Eichenbaum, 2000; Squire et al., 2004; Eichenbaum et al., 2007). This view, however, does not adequately account for behavioral data showing that perirhinal cortical lesions lead to visual discrimination impairments between complex stimuli that share common features (Baxter and Murray, 2001b; Bussey et al., 2003; e.g., Buckley, 2005; Bartko et al., 2007a,b). The observation that lesions of the perirhinal cortex can produce impairments in perception, points to new behavioral assays for assessing the impact of normative aging on the functional integrity of perirhinal cortex.

When cognitive neuroscientists describe perceptual processes they typically refer to the ability of an organism to be aware of and derive meaning from sensory input. On the other hand, memory is the ability to store, retain, and recall information. Paramount to both perceptual and memory processes, however, is the association of different elements of a stimulus or an episode. In this sense it may not be productive to attempt to arbitrarily define when perception ends and memory begins. In fact, because the binding of information represented across disparate areas of the neocortex is believed to be the cornerstone of memory, behavioral deficits resulting from perirhinal cortex lesions can present either as memory or perception impairments depending on the specific task demands.

Murray and Bussey (1999) were the first to formally outline a role for the perirhinal cortex in both perception and memory. The idea is that, as part of a perceptual network, the primary role of the perirhinal cortex is to bind together complex conjunctions of stimulus features (Murray and Bussey, 1999). In support of this notion, perirhinal cortex lesion studies indicate that this region is required for visual discrimination, even in the absence of a memory load (0 delay), if the animal must discriminate between objects that share common features. If the objects have no overlapping features, then other neural systems can presumably participate in the solution of the discrimination and thus behavioral performance on these kinds of tasks can appear normal. For example, when monkeys are trained to discriminate between objects in which no single feature can be used to solve the problem (that is, visual stimuli share features that are predictive of both a correct and incorrect response), animals with perirhinal cortex lesions are impaired relative to controls. On the other hand, perirhinal cortex-lesioned monkeys are able to learn visual discrimination problems if the rewarded and the unrewarded stimuli do not share common elements (Bussey et al., 2002, 2003). Perirhinal lesions also result in performance deficits in rodents on spontaneous object recognition (SOR) tasks as well as in perceptual oddity tasks (Bartko et al., 2007a,b). With respect to sensitivity of these tasks to normative aging, object discrimination (OD) problems can also pose difficulties for older animals. This suggests that these perirhinal cortical-dependent tasks can be used to detect cognitive changes during aging, as described in more detail below.

## Standard spontaneous object recognition (SOR) tasks for rats

Recently our group has examined performance on the standard SOR task with different delays (2 min—24 h), as well as other versions of this task, to assess recognition memory ability in young and old rats (Burke and Barnes, 2010). While there are no behavioral differences observed between age groups on standard SOR tasks in which the objects are distinct and the delays are shorter than 15 min (Cavoy and Delacour, 1993), old rats do show impaired performance at delays greater than this (Bartolini et al., 1996; Vannucchi et al., 1997; Pitsikas et al., 2005; Pieta Dias et al., 2007; Pitsikas and Sakellaridis, 2007; Burke et al., 2010; Hopkins et al., 2011). As is described in more detail below, because of the procedural differences involved in testing the animals on short versus longer delays, it can be inferred that the old rats' inability to discriminate the novel object is not due to “forgetting” over the greater temporal interval, but rather to the older rats behaving as if the novel object is familiar when the procedures do not reduce exposure to extraneous intervening stimuli (Burke et al., 2010).

Additionally, when the objects to be distinguished share common features in the discrimination problem, the older rats are also impaired, even at the shortest delays. This age-sensitive deficit in discrimination, although not as severe, is very reminiscent of the behavior of young animals who have sustained damage to the perirhinal cortex (McTighe et al., 2010). The details of the task procedures necessary to make inferences about the function of this medial temporal lobe structure are outlined below. One technical point needs to be addressed before proceeding, however: for aged rats (and presumably mice) it is very important that before an aged animal goes into OD training, that there is some test of visual function, so that peripheral sensory system changes can be ruled out as a cause of the discrimination problem (this can also be verified by establishing that the older animal shows high performance when the discrimination is conducted at short delays without the animal being removed from the room). One method that we have used is to test rats on the cued version of the Morris swim task as a screen, only including old animals in SOR that show good performance (<1 standard deviation of young mean). For monkeys, we also use an easy (i.e., object pairs do not share any features) OD task as well as detailed ophthalmological exams before proceeding with any testing of perirhinal cortical function.

## Standard spontaneous object recognition (SOR) task procedures

**Figure 1:**
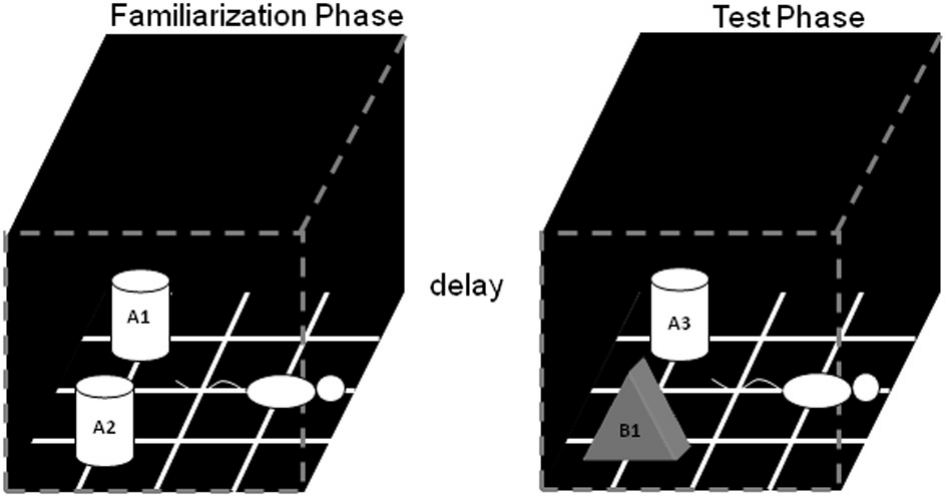
**Schematic of the standard SOR task.** The white cylinders and the black pyramid indicate the location in which the objects were placed during both phases of recognition testing. The schematic white rat shows the orientation in which the rat was placed when it is first put into the arena. During the object familiarization phase (left box), a rat is placed in the arena and allowed to explore duplicate copies of an object (A1 and A2). After this object familiarization phase, the rat is moved out of the arena for a variable delay (2 min, 15 min, 2 h, or 24 h). Following the delay, during the test phase (right arena), the animal is returned to the arena to explore a triplicate copy of the object presented during the familiarization phase (A3) and a novel object (B1) (after Burke et al., 2010).

The general procedure for conducting the SOR task is shown schematically in Figure 1. There are two general phases: the familiarization phase, in which two identical objects are presented to the rat for a 4 min period; and the test phase, in which a copy of the object first seen in the familiarization phase is presented along with a novel object. The total amount of time spent exploring objects during the familiarization phase is typically not different between young and aged rats (Burke et al., 2010, 2011), and can be used to rule out differences in motivation to explore or other motor impairments that may confound comparisons of young and aged rat SOR task performance. The natural tendency of mammals is to explore the novel object more in the test phase than the familiar object, and the difference in exploratory behavior has typically been taken to be a measure of memory for the familiar object (but see the arguments for potential confounds below).

### Detailed methods

Within two weeks of visual acuity being assessed using Morris swim task procedures, rats complete the SOR task. The apparatus used for the task is a box constructed from wood, 30 cm by 30 cm, with walls that are 30 cm tall (Figure 1). All walls of the apparatus are painted black, and the floor is black with a grid used to ensure that the location of objects do not change between object familiarization and test phases. Before recognition testing begins the rats habituate to the arena for 10 min on two sequential days. Recognition testing begins on the third day. It is also very important that rats only perform 1 trial/day, otherwise their exploratory behavior can habituate and the data can become unreliable.

An overhead camera and a video recorder, or a digital camera/computer, are used to monitor and record the animal's behavior for subsequent analysis. The episodes of exploration are analyzed by defining “exploratory behavior” as the amount of time that the animal directs its nose toward the object at a distance of ~2 cm or less (Ennaceur and Delacour, 1988). Any other behavior, such as resting against the object, or rearing on the object is not considered to be exploration. Exploration is scored by an observer blind to the rat's age and to the duration of the delay between the object familiarization and the test phases. Additionally, the amount of time spent exploring objects during the test phase is scored prior to measuring the amount of exploration during the object familiarization phase. This reverse-order of analysis ensures that the scorer is blind with respect to which object is familiar and which object is novel. Finally, the positions of the objects in the test phases, and the objects used as novel or familiar, are counterbalanced between the young and the aged animals in all conditions of the OD experiments.

As just reviewed, there are two components or “phases” of SOR testing: Familiarization and Test. For the standard SOR task, rats participate in four object familiarization and test phases with four different delays (2 min, 15 min, 2 h and 24 h) between familiarization and test. Each rat participates in every delay condition once. The order of the delays are shuffled (i.e., Burke et al., 2010) individually for every rat. Again, the stimuli presented are triplicate copies of objects made of glass, plastic, or wood that varied in shape, color, and size. Therefore, different sets of objects are texturally and visually unique. In this variant of the task all rats are exposed to the same stimulus sets for a single delay condition. This is not the case in the version of the SOR that measures the effect of perceptual difficulty on object recognition (see below), in which the delay is 30 s for two different trials and the similarity of the objects pairs is manipulated.

In the object familiarization phase, duplicate copies of an object (in Figure 1, A1 and A2) are placed 10 cm from each adjacent wall. The white cylinders and the gray pyramid indicate the exact positions of the objects for both phases of recognition testing. To reduce variability in behavior within and between rats, it is important to always place the animal into the arena in the same orientation, facing the center of the wall opposite to the objects (Figure 1, schematic white rat). The rat is allowed a total of 4 min of exploration in the open arena. After the object familiarization phase, delays are imposed before exposure to the box in the test phase. For the 2 min delay, the rat is placed in a covered pot next to the apparatus. This prevents the animal from being exposed to extraneous visual stimuli. For the 15 min, 2 h, and 24 h delays, the rat is returned to its cage in the colony room. Rats are placed in covered pots for all transportation between the colony room and the experimental apparatus, although this does not eliminate exposure to more intervening stimuli in the longer delay conditions compared to during the 2 min delay, where they are not transported (discussed more below).

During the test phase, the animal is returned to the apparatus and placed back into the same start location as for the object familiarization phase. Again, the rat is allowed 4 min of exploration but is presented with two different objects than had been used during the familiarization phase. One object (Figure 1, A3) is the third copy of the triplicate set of the objects used in the object familiarization phase (familiar), and the other is a novel object (Figure 1, B1). All objects and the apparatus are washed with 70% ethanol between every trial and before procedures begin with another rat. Different sets of objects are used for each delay condition, and each object is trial-unique.

**Figure 2:**
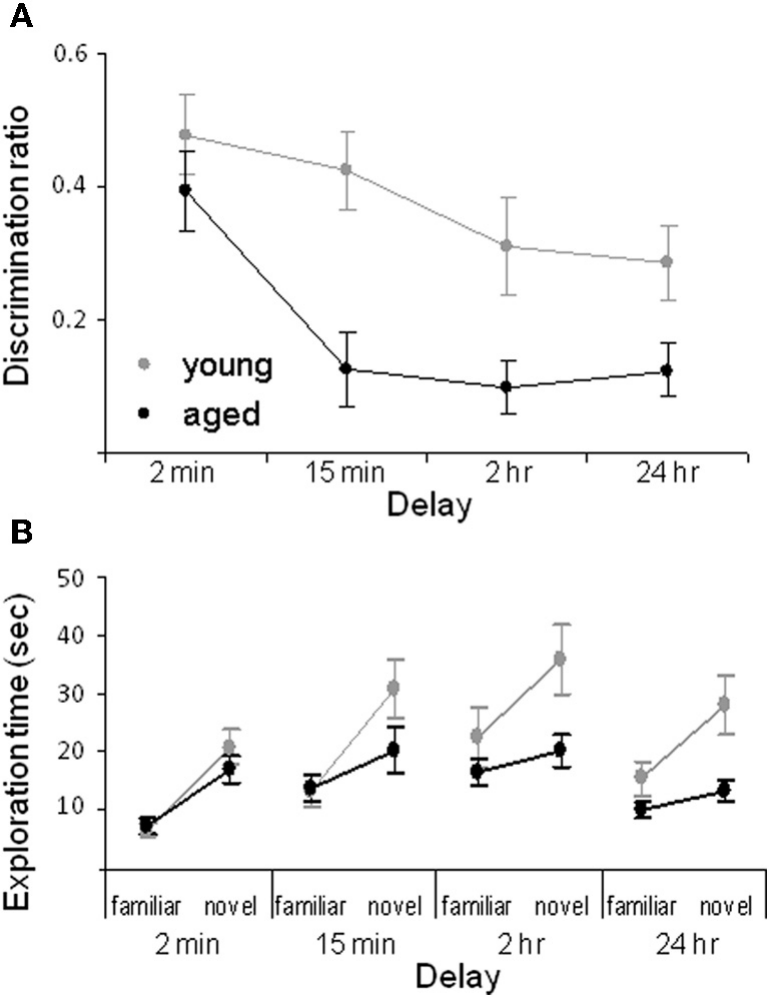
**Spontaneous object recognition task performance in young and aged rats. (A)** The mean discrimination ratio of the adult (green) and the aged rats (purple) during the test phase for the four different delay conditions. A higher discrimination ratio indicates that the animal spent more time exploring the novel object relative to the familiar object. **(B)** The mean amount of time young (green) and aged (purple) rats spent exploring the familiar and the novel object during the test phase for the four different delay conditions. Error bars represent ±1 standard error of the mean (after Burke et al., 2010).

We have shown that aged rats are impaired at the standard SOR task when the delay interval between the sample and the test phase is at least 15 min. Particularly, for the 15 min, 2 h and 24 h delays the aged rats have a significantly lower discrimination ratio compared to the young animals (Figure 2A). The discrimination ratio is calculated by dividing the difference in exploration times between the novel and familiar objects by the total amount of object exploration time during the test phase of the SOR task (Dix and Aggleton, 1999). Therefore, a discrimination ratio of zero indicates that the rats did not show an exploratory preference for the novel objects, while increased novel object exploration would produce higher values. Although lower discrimination ratios are often interpreted as the animal “forgetting” what object was encountered during the familiarization phase, a decrease in the discrimination ratio can occur either from increased exploration of the familiar object or reduced exploration of the novel object. Thus, the raw exploration times of the novel and familiar objects during the test phase must also be compared between age groups. Analysis of the raw exploration times of individual objects, during the test phase, shows that the age-associated reduction in the discrimination ratio at the 15 min, 2 h, and 24 h delays is associated with a reduction in novel object exploration in the aged compared to the young rats. In contrast, the amount of time spent exploring the familiar objects is similar in both age groups (Figure 2B).

The data suggest that the primary age-related deficit in object recognition at longer delays arises from old rats behaving during the test phase as if the novel object is familiar rather than from forgetting. Because reduced novel object exploration is not observed in aged rats after short delays and when objects are first encountered during the familiarization phase, it is possible that this age-associated deficit is due to the intervening stimuli encountered during long delay periods. Specifically, the 2 min delay of standard SOR task is the only condition in which the rats remain in a covered pot in the same room as the testing arena for the delay. Therefore, in this condition the amount of extraneous stimuli that the rat encounters before the test phase is reduced relative to the other conditions. This suggests that aged rats have been influenced by stimuli that are spontaneously encountered during longer delay periods. Conceivably some of these extraneous stimuli share common features with the objects presented during the test phase. Because old animals may be less able to discriminate different stimuli that share common features, the aged rats identify the novel object as familiar. Therefore, one could predict that if an aged rat was exposed to more stimuli during the 2 min delay period, then novel objects would be identified as familiar even at this short delay. In fact, this was observed. When rats are moved to a new room, but the delay is only 2 min, aged rats show less novel object exploration relative to young animals (Burke et al., 2010). These data indicate that rats must be removed from the testing room for object recognition deficits to manifest, and also suggests that aged rats should have difficulty discriminating between similar stimuli. This hypothesis has been confirmed with the task described below (Burke et al., 2011).

## Procedures for spontaneous object recognition (SOR) task using similar objects

Because it is clear that the object recognition deficit in aged rats is largely dependent on interference effects (i.e., whether intervening stimuli are experienced between familiarization and test conditions), versions of the SOR task that have short delays but use objects with overlapping features (i.e., increased perceptual difficulty) have been implemented, and have been extremely informative with respect to the age differences observed. In the variant of the task in which the objects to be discriminated are visually similar, old rats can show decreases in OD performance even at a short delay (30 s; Burke et al., 2011). Again, these data from aged rats, while perhaps not as severe, are at least similar to the kinds of deficits observed in young animals with perirhinal cortical lesions (Bartko et al., 2007a,b).

### Detailed methods

This variant of the SOR task is identical to the task described above with two exceptions. First, rats participate in only two trials and both trials have the same 30 s delay. Rather than the delay changing, the amount of similarity between the objects to be discriminated during the test phase is the independent variable. Second, the behavioral apparatus is different. This enables the same rats to participate in both tasks by reducing the degree to which the animals habituate to exploring objects in a single environment.

**Figure 3:**
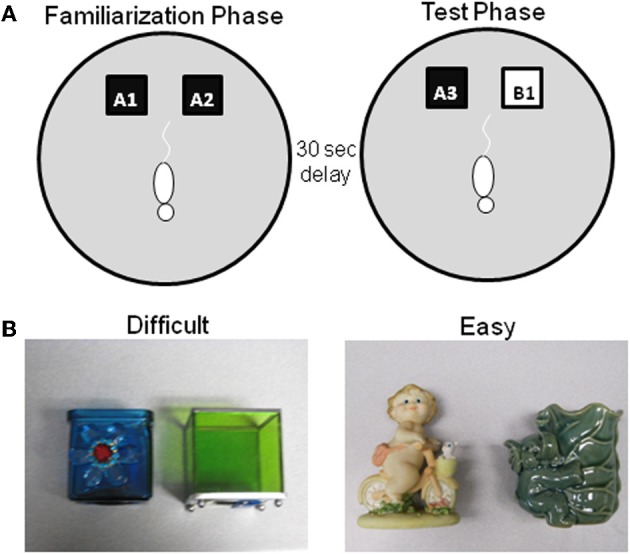
**Schematic of spontaneous object recognition task using objects with similar features. (A)** The testing arena is a circle ~201 cm in circumference with a wooden floor and 40.6 cm high walls. The black squares indicate the location in which that the objects are fixed in place during the familiarization phase (left arena). The schematic white rat is shown in the initial orientation of the rat when it is first placed in the arena. In the object familiarization phase (right arena), a rat is placed into the arena to explore duplicate copies of an object (A1 and A2). The rat is then moved from the arena and placed into a covered pot adjacent to the arena for a 30 s delay. Following the delay, during the test phase (bottom arena), the animal is returned to the arena to explore a triplicate copy of the objects presented during the familiarization phase (A3) and a novel object (B1). **(B)** Example objects used for the two trials of this task, both of which have a 30 s delay between the familiarization and test phases. In one condition the objects are similar to each other (Left panel–Difficult), while in the other condition the objects are distinct and share no features (Right panel–Easy) (after Burke et al., 2011).

The apparatus used for this experiment is a circular arena ~201 cm in circumference with a wooden floor and 40.6 cm high walls that are constructed from stiff black poster board (Figure 3). Again the animals' behavior is recorded for subsequent offline analysis of “exploratory behavior,” which is defined as the animal directing its nose toward the object at a distance of ~2 cm or less (Ennaceur and Delacour, 1988). Any other behavior, such as resting against the object, or rearing on the object is not considered to be exploration.

As in the standard SOR task, before testing begins, all rats are exposed to the empty apparatus for 10 min on two consecutive days. Recognition testing begins the day immediately following this habituation procedure. All rats participate in two object familiarization and test phases with two different levels of perceptual difficulty (“Easy” and “Difficult”). In the Easy condition the test objects do not share any common features, that is, they are different in shape, color, and texture, although they are similar in overall size. For the Difficult condition, the test objects are both cubic in shape, and 3 of the 4 sides of the objects have a similar smooth texture. Therefore, in the Difficult condition the test objects have more features in common relative to the Easy condition (Figure 3 shows sample objects). A previous experiment has used objects constructed from LEGOs® to assess the effects of the feature overlap on SOR task performance in Lister hooded rats (Bartko et al., 2007a). Although LEGOs® have been used in pilot studies, reliable recognition performance has not been achieved in F344 rats in either age group with these stimuli, for which we have no definitive explanation. Therefore, 3D junk objects are used as stimuli, for which high levels of recognition performance in young rats can be achieved (Burke et al., 2011). For both the Easy and Difficult conditions rats are tested with one of two different sets of objects. The order that a rat participates in each condition and the location of the novel object (left or right) is pseudo-randomized individually for every rat. Additionally, the objects used for the familiarization phase and the novel object are also counterbalanced to control for possible differences in object preference.

**Figure 4:**
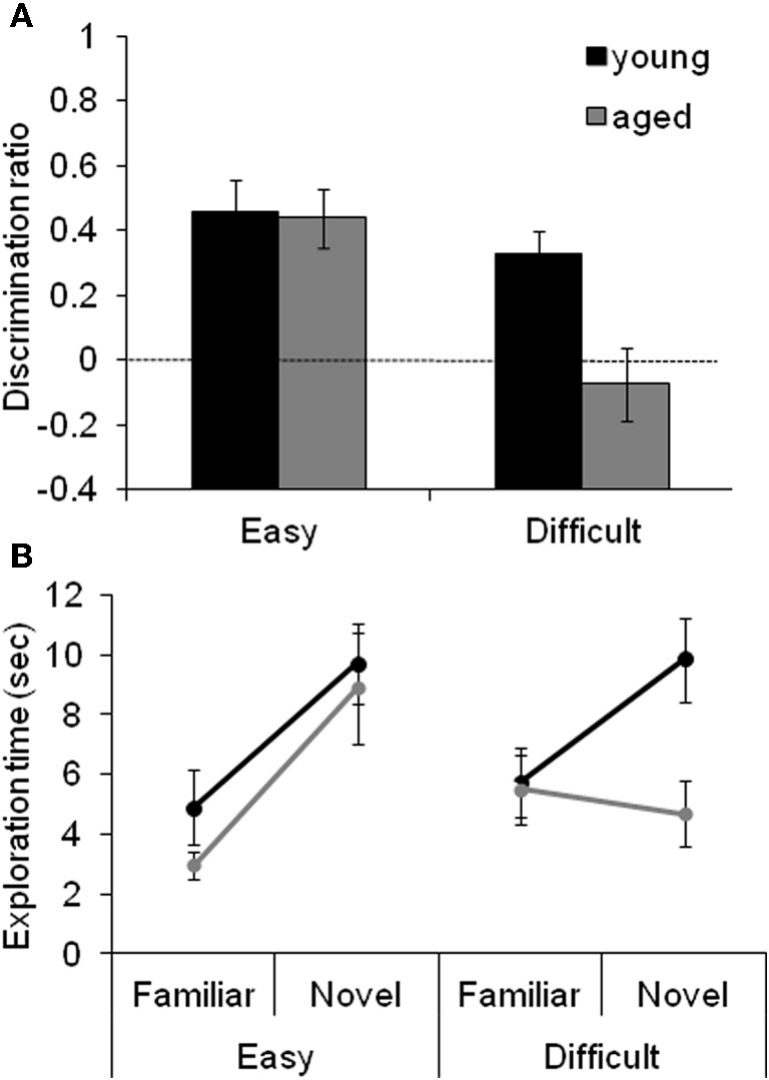
**The effect of difficulty on SOR task performance. (A)** The mean discrimination ratio of the adult (black) and the aged rats (gray) measured during the test phase following a 30 s delay for the “Easy” and “Difficult” conditions. A higher discrimination ratio indicates that the animal spent more time exploring the novel object relative to the familiar object. The aged rats had significantly smaller discrimination ratios when compared to the adult rats for the Difficult condition, but not for the Easy condition. The dashed horizontal line indicates chance performance. **(B)** The mean amount of time young (black) and aged (gray) rats spent exploring the familiar and the novel object during the different testing conditions. Compared to the young animals, the aged rats spent significantly less time exploring the novel object during the test phase of the Difficult condition. In contrast, novel exploration between age groups was similar during the Easy condition. Error bars represent ±1 standard error of the mean (reprinted with permission Burke et al., 2011).

Figure 4 shows the data obtained from young and aged rats on the SOR task with “Easy” and “Difficult” discriminations. These data indicate that the age-associated reduction in the discrimination ratio for the Difficult condition selectively results from the aged rats exhibiting reduced exploration of the novel object when it shares features with the familiar object. The observation that there is a significant reduction in the amount of exploration time of the novel object in the test phase of the Difficult condition relative to the exploration time of a novel object in the familiarization phase provides additional support for this hypothesis.

## Perirhinal cortical function in non-human primates: two-choice object discrimination (OD) testing

**Figure 5:**
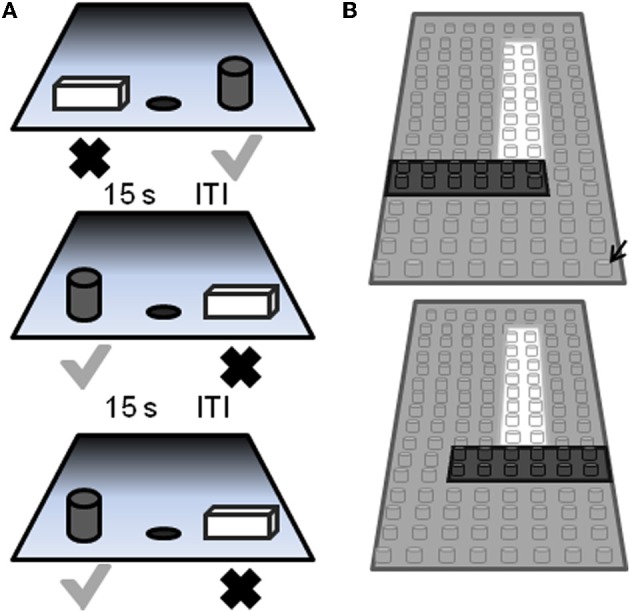
**Schematic of the two-choice object discrimination (OD) apparatus and procedure. (A)** Examples of three different OD task trials. For each OD problem, an object is placed over the left and right food well. The gray cylinder represents the rewarded object (gray check) and the other object of the pair (rectangular cube; black X) was never rewarded. A 15 s inter-trial interval (ITI) is imposed between successive trials, and the side of the rewarded object pseudo-randomly changed across trials. **(B)** A schematic of the pair of LEGO® objects with 86% feature overlap. Feature overlap is calculated by dividing the number of LEGO® “pips” (indicated by the black arrow) that were the same between the two objects by the total number of bits in a single object (reprinted with permission Burke et al., 2011).

Perirhinal cortical function in monkeys can be tested using a standard OD task in which the amount of feature overlap between the two objects can be varied (Figure 5). Aged monkeys are just as capable of performing this task as are young animals when the objects to be discriminated are very different. When the objects are constructed from LEGO®, however, aged monkeys perform worse relative to young if the objects have significant feature overlap (Burke et al., 2011), as discussed below.

### Detailed methods

The behavioral apparatus and testing procedures used in this OD task have been described in detail previously (Rapp, 1990; Bachevalier et al., 1991; Rapp et al., 2003). Briefly, a modified Wisconsin General Test Apparatus (WGTA; Harlow and Bromer, 1938) is used for all behavioral testing. The WGTA is composed of a chamber with vertical bars situated in front of a tray for stimulus presentation. The tray includes three equally spaced wells. Either the left or the right well is baited with a food reward during testing, and the middle well is not used during OD testing. A wooden guillotine door, controlled by the experimenter, is used to limit the animal's physical access to the wells and to impose 15 s inter-trial intervals (ITIs). A one-way mirrored screen allows the tester to remain undetected while observing the animal's performance.

**Figure 6:**
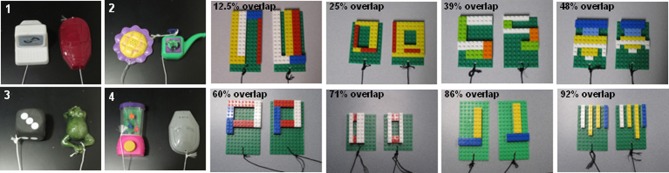
**Test stimuli used of object discrimination in monkeys. 1–4** are examples of the distinctly different objects used in Easy Condition of the Object Discrimination Task. Subsequent photographs are of all eight LEGO® object pairs in the Difficult Condition, ranging in overlap from 12.5 to 92% (after Burke et al., 2011).

On each OD trial, subjects choose between two objects, one of which is consistently associated with reward across trials (Figure 5A), but the location of the rewarded object (left versus right) is pseudo-randomly changed for each trial. The same discrimination problem is presented for 30 trials per day for successive days until the animal selects the rewarded object with 90% accuracy. If this performance criterion is achieved on the first day of testing, the animal performs one additional day of testing with the same object pair. Following the achievement of criterion performance, or the second day of testing if this is achieved on day 1, a 48 h delay is imposed before a final 30-trial session. Animals are tested on 12 successive discriminations problems according to this schedule. For the first four discrimination problems object pairs are visually distinct junk objects that do not share any common features (Figure 6, part 1, 2, 3, 4). The other eight object pairs are constructed from LEGOs® (Figure 6, constructed to have 12.5–92% overlap) so the experimenters can manipulate the amount of “feature overlap” between pairs (Bartko et al., 2007b). Feature overlap is calculated by dividing the number of LEGO® “pips” (indicated by the black arrow in Figure 5B) that are the same between the two objects by the total number of pips in a single object. For this example each object contained 128 LEGO® pips and the there were 96 pips in common. The percent of overlap for the 8 LEGO® objects ranged from 12.5 to 96% (Figure 6), and the order of presentation is pseudo-randomly shuffled for each monkey individually.

The OD task described above, in which the object pairs do not share any features, has been used in our lab to show that both young and old animals are able to achieve 90% correct performance criterion within two days of testing. Moreover, there is no significant age group difference in the mean percent correct for selecting the rewarded stimulus when the object pairs are very different (Figure 6: 1–4). This is consistent with previous data (e.g., Bachevalier et al., 1991; Lai et al., 1995), which indicate that there is no difference in the ability of young and aged monkeys to learn which of two objects is associated with reward when the objects do not contain similar features.

**Figure 7:**
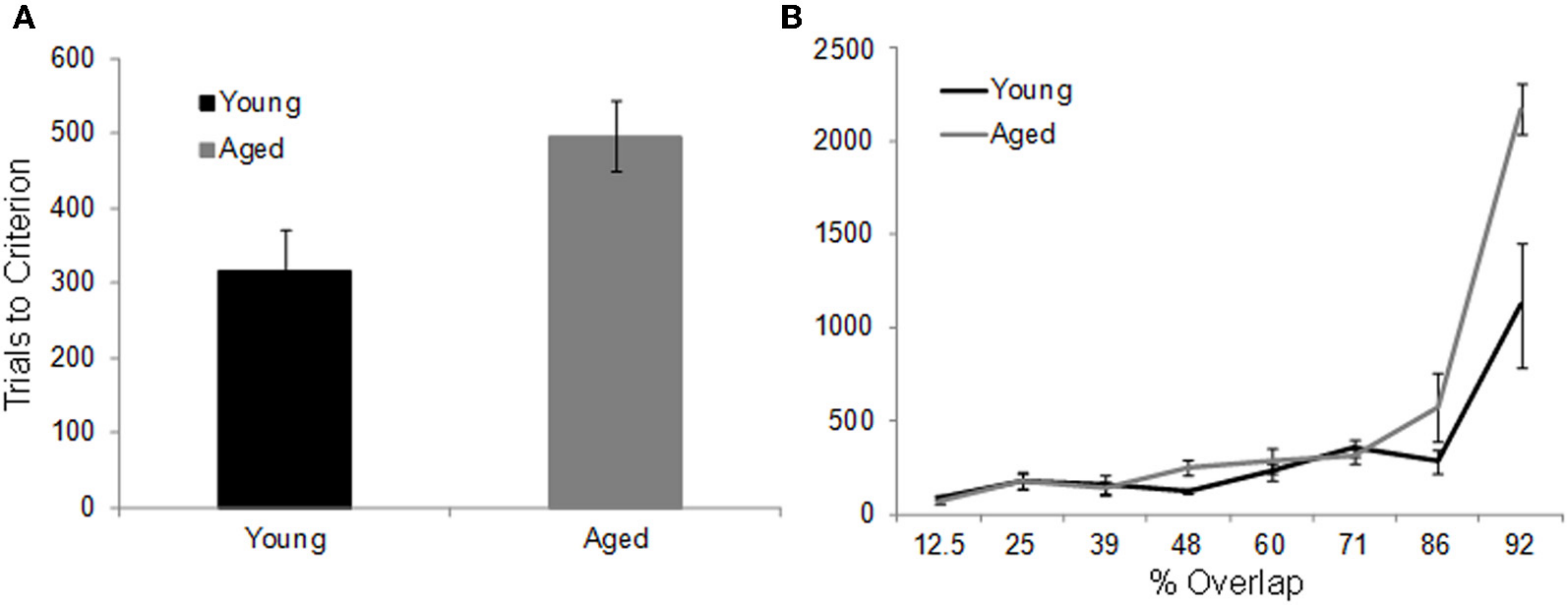
**The effect of feature overlap on two-choice object discrimination (OD) task performance. (A)** The mean number of trials required for young (black) and aged (gray) monkeys to reach performance criterion of 90% correct on the two-choice OD task when the object pairs shared features. It took the aged monkeys significant more trials to learn which object was rewarded. **(B)** The mean number of trials to criterion (Y-axis) across the different levels of feature overlap (X-axis). There was a significant exponential relationship between percent overlap and trials to criterion in both the young and the aged monkeys. Relative to the young animals, the aged monkeys required significantly more trials to reach criterion on the 86% and 92% overlap conditions. Error bars represent ±1 standard error of the mean (reprinted with permission Burke et al., 2011).

In contrast to the standard OD task, when the object pairs are constructed from LEGOs® so that they share features, it takes the aged monkeys more trials to reach criterion performance compared to the young animals. Figure 7A shows the mean number of trials that the young (black) and the aged (gray) monkeys required to reach a performance of 90% correct. Figure 7B shows the mean of the number of trials needed to reach criterion performance as a function of percent overlap for both age groups. It is evident from this plot that as the degree of overlap increased, more trials are required for an animal to learn which object of the pair was associated with a reward (Burke et al., 2011).

**Figure 8:**
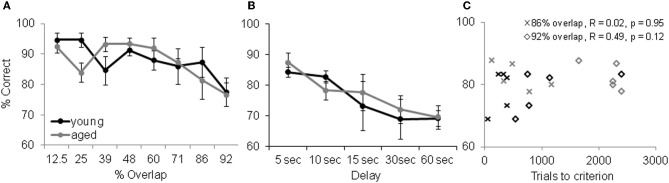
**Memory and Object discrimination (OD) task performance. (A)** OD task performance following a 48 h delay. The percent correct (Y-axis) over 30 trials for the different levels of feature overlap (X-axis). After the two-choice OD was acquired, the young (black) and aged (gray) monkeys did not perform significantly differently from one another following the delay (reprinted with permission Burke et al., 2011). **(B)** Performance of the delayed response task. Percent correct (Y-axis) across the different delay conditions (X-axis) shows that working memory was comparable between this population of young and aged monkeys. Error bars represent ±1 standard error of the mean. **(C)** OD task performance (X-axis) versus DR task performance (Y-axis, 10 s delay) shows that there was no significant relationship between working memory ability and a given monkey's ability to learn difficult discrimination problems.

Although increasing the percent of feature overlap leads to an age effect in the ability of monkeys to acquire an association between an object and reward, once this association is learned, there is no age difference in the ability of monkeys to remember that association following a 48 h delay. Figure 8 shows the percent correct for the 30 trials that followed the 48 h delay after the performance criterion was achieved for the young (black) and aged (gray) monkeys. After the two-choice OD is acquired, the young and aged monkeys do not perform significantly different following the delay. These data indicate that there is a dissociation between the effects of feature overlap on the ability to discriminate between two stimuli and the ability to remember that discrimination (Burke et al., 2011).

Although there is a dissociation between the ability to visually discriminate and remember high feature overlap problems, it is conceivable that differences in selective attention or working memory between young and aged monkeys contribute to the results discussed above. It has been shown, however, that in the absence of interference aged subjects are just as able to selectively attend to a stimulus as young subjects (e.g., Gazzaley et al., 2005). Thus, we do not think that attention deficits contribute to performance differences between age groups on the OD task. Aged humans and other animals do, however, have deficits in working memory that could contribute to the visual discrimination impairment observed here. Specifically, more difficult discrimination problems could require the animal to look between the objects while holding feature information in working memory. To address this issue, we compared performance on the OD task at the two highest levels of feature overlap (86% and 92%) to performance on the delayed response (DR) task. The DR task is a classic test of spatial working memory that shows age-related declines in performance (e.g., O'Donnell et al., 1999; Alexander et al., 2008). In this task the monkey is required to remember if a reward is located at one of two different spatial locations. Importantly, the colony of monkeys that completed the OD task did not show a significant age difference in DR task performance at delays from 5 to 60 s (Figure 8B). Moreover, there was not a significant relationship between DR task performance at any delay and OD task performance at the 86% and 92% overlap conditions. Figure 8C shows a scatter plot of OD task performance for the two highest overlap conditions plotted against DR task performance at the 10 s delay. These observations rules out differences in working memory as a potential explanation for the age-associated difference in OD task performance at the 86% and 92% feature overlap conditions.

## Perirhinal cortex and feature conjunctions: converging evidence from humans

Recent studies in humans with brain lesions support the animal work suggesting that perirhinal cortex is necessary for processing complex conjunctions of features within objects. A common finding across studies is that patients with damage to the medial temporal lobe extending into perirhinal cortex are impaired at various types of OD tasks when the objects have a high, but not low, number of overlapping features. In contrast, patients with medial temporal lesions that are confined to the hippocampus perform equivalently to appropriately matched controls on OD tasks regardless of the level of feature ambiguity (Barense et al., 2005, 2007; Lee et al., 2005; Taylor et al., 2007; Barense et al., 2010a; Lee and Rudebeck, 2010).

**Figure 9:**
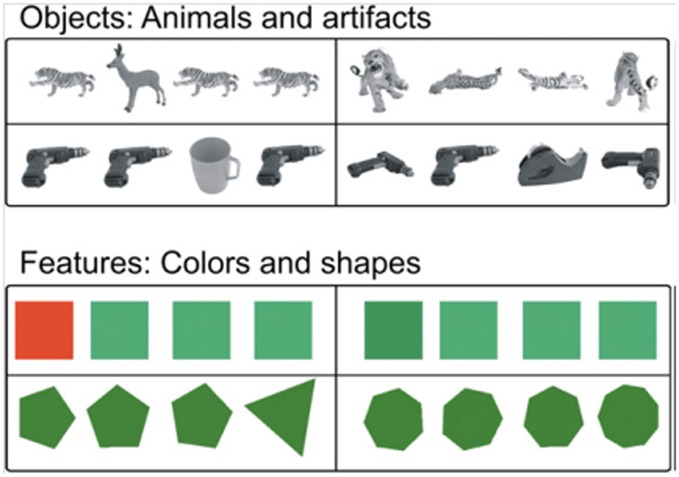
**Example stimuli from Devlin and Price (2007).** Easy (left column) and difficulty (right column) trials are shown for common objects (top rows) and feature stimuli (lower rows). This task is almost identical to visual arrays used by Buckley et al. (2001) with non-human primates, facilitating cross-species comparisons (reprinted with permission Devlin and Price, 2007).

Functional neuroimaging studies with healthy young participants provide additional support for these findings (Devlin and Price, 2007; Lee et al., 2008; O'Neil et al., 2009). In a positron emission tomography study, Devlin and Price (2007) used a perceptual discrimination task originally developed for monkeys (Buckley et al., 2001) that included both objects and feature stimuli with two levels of difficulty (Figure 9). Participants chose the non-matching item out of an array of four objects. For the difficult object matching condition, the three matching objects were shown from different viewpoints. The task can only be solved by integrating multiple visual features to identify a single object that is presented from different views. Both the easy and difficult feature conditions can be solved by matching a single feature (color or shape). Perirhinal activation was observed above baseline when the objects were shown from different viewpoints, but not for the simple object matching condition, or for either the easy or hard feature matching conditions. Taken together with more recent functional MRI work (Barense et al., 2010b, 2011), the study established a similar perceptual specialization for perirhinal cortex across species, suggesting that this region in critical for the integration of visual features into an abstract, view-invariant, representation.

Additionally, it appears that perirhinal cortex may be part of a network of anterior temporal regions that respond to the amodal semantic representations of objects (Murray and Bussey, 1999) regardless of whether the stimuli are presented as spoken words, written words, or pictures (Visser et al., 2010). Given the widespread and polysensory afferents to this region (Suzuki and Amaral, 2004), perirhinal cortex is well placed to act as an interface between perceptual processing and semantic knowledge, supporting the interactive activation of surface representations across different modalities (Binney et al., 2010).

**Figure 10:**
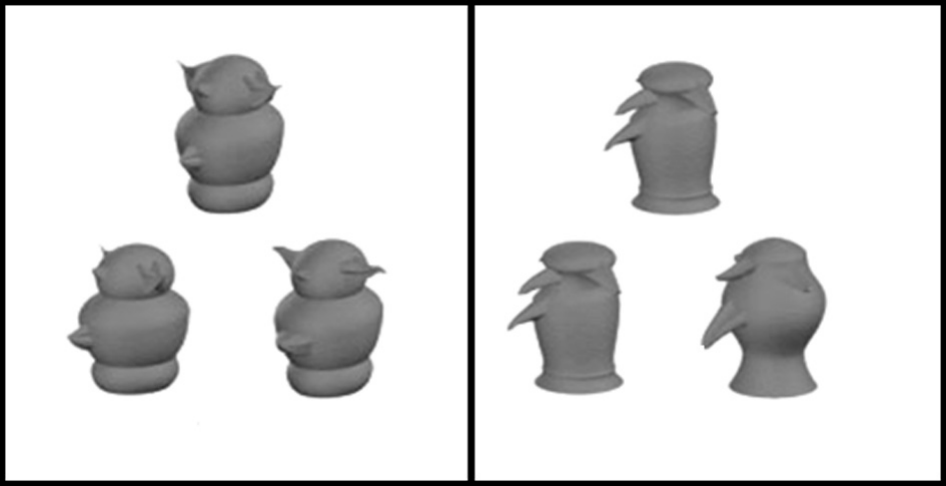
**Novel objects (greebles) used by Barense and colleagues (2010b).** Participants were told that of the three pictues presented per trial, two depicted the same object, whereas the third picture was of a different object. They were instructed to select the different stimulus. In the difficult condition, the two matching objects were shown from two different viewpoints, while in the easier condition (right panel) the matching objects were shown from the same viewpoint (adapted from Barense et al., 2010b).

Given that most OD tasks involve pictures of common objects or animals (e.g., Devlin and Price, 2007), it could be argued that increased perirhinal activity reflects retrieval of a stored semantic representations for the object, rather than perceptual processing *per se*. To address this question, Barense et al. (2010b) manipulated viewpoints in a discrimination task involving unfamiliar faces and novel objects (greebles) in a functional MRI study (Figure 10). They found increased perirhinal activity when unfamiliar faces and objects were shown from different viewpoints compared to the same viewpoints. In contrast, the posterior hippocampus showed increased activity when viewpoint was manipulated for faces and complex scenes, but not novel objects. In a related study, Barense et al. (2011) used an OD task that directly varied the meaningfulness of the stimuli, comparing well-known faces to novel faces, and everyday objects to novel objects (greebles). They found that meaningful, relative to novel, faces, and objects increased fMRI activation within the perirhinal cortex and hippocampus. Importantly, the increased activation due to meaningfulness was evident even when the faces and objects were not well remembered later on, suggesting that the difference between meaningful and novel stimuli reflects a combination of perceptual and conceptual processes rather than incidental encoding into long-term memory. These two studies provide convergent evidence that the medial temporal lobe is involved in processing that goes beyond long-term declarative memory (either episodic or semantic) and suggests a critical role for perirhinal cortex in integrating complex perceptual features of faces and objects into view invariant, abstract representations.

Nonetheless, most researchers agree that the perirhinal cortex likely contributes not only to perceptual but also mnemonic functions in humans (Murray and Bussey, 1999; Devlin and Price, 2007; Barense et al., 2011; Burke et al., 2011). Encoding, conceptual processing, and perceptual processing are not entirely independent processes. As Barense and colleagues (2011) point out, the notion that more extensive processing of both perceptual and conceptual aspects of an experience leads to better memory has a long history in cognitive psychology (Craik and Lockhart, 1972; Craik and Tulving, 1975; Craik, 2002). It should not be surprising, therefore, that the same regions involved in perceiving complex stimuli are also involved in remembering them.

## Human aging and perception: tasks for evaluating perirhinal involvement

It has long been hypothesized that age-related changes in cognitive abilities and sensory/perceptual functioning are connected (e.g., Lindenberger and Baltes, 1994; Baltes and Lindenberger, 1997; Park et al., 2004; Ghisletta and Lindenberger, 2005; Nagel et al., 2007), although some recent longitudinal studies suggest that this association is somewhat weaker than originally suggested by earlier cross sectional data (Anstey et al., 2003; Lindenberger and Ghisletta, 2009). By this view, as basic and more coordinated sensory and perceptual mechanisms become less efficient, and more prone to error with advancing age, perceptual task demands may call for additional executive control mechanisms, such as sustained attention and inhibitory control. This compensation for decreases in sensory and perceptual functioning results in increasing demands on executive control mechanisms, which are themselves negatively impacted with advancing age (Craik, 1983; Park and Reuter-Lorenz, 2009; Craik and Rose, 2011).

Although alterations in the central processing of visual and auditory information have been shown to account for a significant amount of age-related variance on cognitive tests including speed of processing, memory, verbal fluency, and reasoning (Baltes and Lindenberger, 1997; Lindenberger and Baltes, 1997), the neuroanatomical bases for these age-related sensory changes remains relatively unexplored. One theory (Li and Lindenberger, 1999; see also Braver et al., 2001; Nieuwenhuis et al., 2002) suggests that many age-related cognitive changes can be attributed to changes in dopaminergic neuromodulation, resulting in less distinct neuronal signaling, less separable processing pathways, and less differentiated cerebral representations (Andrews-Hanna et al., 2007; Park and Reuter-Lorenz, 2009).

Another area of recent interest, directly relevant to the present discussion, is age-related changes in the efficiency with which older adults perceive complex objects. For example, Park et al. (2004) demonstrated age-related decreases in selectivity of the ventral visual cortex to four categories of visually presented stimuli. In contrast to older adults, young adults showed more distinctive activation patterns that represented a more differentiated neural representation for each class of stimuli. Park and colleagues (2004) suggested that the reduced uniqueness of stimulus representations could play an important role in age-related degradation of speed of processing, particularly perceptual speed. Other studies have focused on specific subregions of the ventral visual stream. Using an adaptation paradigm, Chee et al. (2006) showed objects, scenes, and objects embedded in scenes, to older adults and young adults. Older adults, relative to young adults, showed a loss of adaptation to repeated objects presented in the context of changing backgrounds but not to repeated objects presented in isolation. They interpret this result to indicate that older adults are deficient in concurrent visual processing of objects and scene backgrounds. The authors suggest that at least some of the age-related impairment observed during associative encoding (Sperling et al., 2003) might be explained by the tradeoff between attending to contextual information at the expense of object processing. Incomplete or inefficient processing of objects may result in insufficient information to complete the binding operation between objects and context in medial temporal regions, resulting in poor subsequent memory for objects and their contexts.

**Figure 11:**
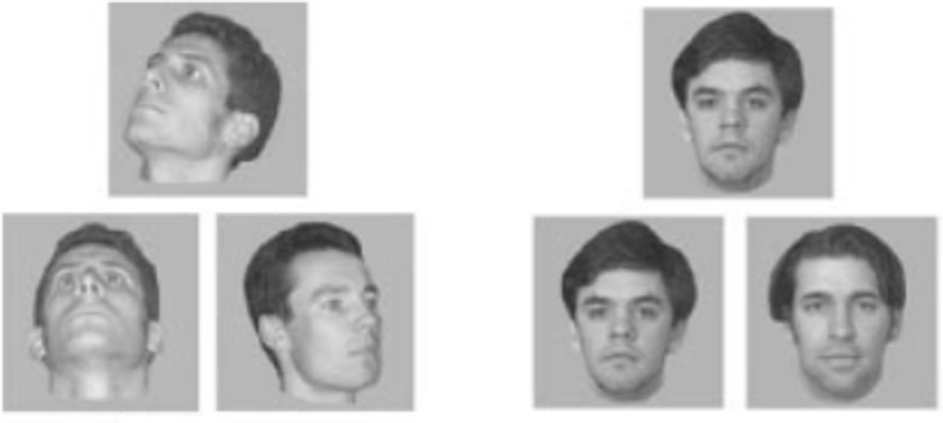
**Stimuli from the face matching task used in Barense et al. (2010b).** In the right panel, the matching faces are presented from the same viewpoint, while on the left, the matching faces are presented from different viewpoints, thus requiring the comparison of multiple features. Similar viewpoint-variant faces are used in the Benton Test of Facial Recognition (Hamsher et al., 1979) (adapted from Barense et al., 2010b).

To date, no studies comparing younger and older adults have been completed using identical stimuli and paradigms previously shown to engage the perirhinal cortex in neuroimaging studies of humans (Devlin and Price, 2007; Barense et al., 2010a,b, 2011). Although there is evidence that older adults are inefficient in processing objects, it remains to be seen whether some of that inefficiency arises because of age-related functional changes to the perirhinal cortex. However, one time-honored neuropsychological test that is virtually identical to the novel face matching tests used by Barense and others provides some preliminary evidence. The Benton Test of Facial Recognition is actually a face discrimination task requiring the identification of one or more unfamiliar faces in a match-to-sample multiple choice display (Hamsher et al., 1979). On each trial, the target picture is a black and white front-view photograph of an unfamiliar face. The match-to-sample choices include the identical front-view photograph, photos of the same face taken from off-angle viewpoints, or front-view photos taken under different lighting conditions. The matching conditions from differing viewpoints and possibly using different lighting conditions are almost identical to the task used in Barense et al. (2010b; Figure 11). As far back as 1983, Benton and colleagues (Eslinger and Benton, 1983) showed that from ages 65 to 94, face matching performance in older adults declined by an average of 0.5 standard deviations per decade, with a regression coefficient for age of –0.51, *p* < 0.0001. Unfortunately the performance measure for the test combines easier identical match trials with the more difficult viewpoint-variant and lighting-variant conditions. Based on the literature described above, these latter trials, in particular, should rely on perirhinal cortical processing. More direct evidence is needed on facial and OD tasks in older adults that can compare directly such high ambiguity conditions to conditions that can be judged based on a single or small set of features.

## Summary and overall conclusions

Recent experiments have revealed that both rats and monkeys have age-associated impairments in perirhinal-dependent perception, that is, both species have a reduced ability to discriminate between similar stimuli in advanced age. There is also a significant literature that suggests that damage to the perirhinal cortex in humans results in similar defects in OD, when objects have a high level of feature overlap (Barense et al., 2007). This clearly suggests that assessing perirhinal-cortical function in elderly humans is an important research avenue to explore, and points to this brain structure as a potential therapeutic target for alleviating some aspects of age-related cognitive decline. Additionally, because of the close correspondence between paradigms that have been used successfully to engage perirhinal cortical function in rats, monkeys, and humans, this area of research lends itself particularly well to cross-species studies of age-related changes in OD and the impact that such perceptual impairments may have on memory function.

### Conflict of interest statement

The authors declare that the research was conducted in the absence of any commercial or financial relationships that could be construed as a potential conflict of interest.
